# Supplementary material for: Encouraging pro-environmental behavior: Do testimonials by experts work?
Source: PLoS One. 2023 Oct 4;18(10):e0291612. doi: 10.1371/journal.pone.0291612 (PMC10550155; doi:10.1371/journal.pone.0291612)
Supplement: S1 Appendix — (DOCX) [file pone.0291612.s001.docx]

**S1 Appendix A. Transcript of the video testimonial provided by an expert**

Doug Tallamy, Entomology and Wildlife Ecology: The ability for ecosystems to function in our managed landscapes and our urban-suburban matrix is very poor because we have landscaped primarily for looks. We have treated plants as if they were decorations and forgotten all about their ecological roles. So, we have sacrificed the ability of all of the land we use for our suburban neighborhoods to make ecosystem services that not only support other creatures, but they support us as well. If you produce plants that insects cannot reproduce on and develop on, you are creating an inoperative ecosystem. What this homeowner has done is put these plants back in the suburban landscape. Most of these plants are indigenous. They are native to this area, which means they are important components of the food webs that originally were in this area. So, you have lots of wildlife because these plants are filtering our water and sequestering carbon, they are supporting the food webs, they are supporting the pollinators, they are producing oxygen. All of the ecosystem services the plants provide are now happening right in this yard because it is not one vast lawn. I care about this because we need these other living things to run the ecosystems that we depend on as humans. So, we can think of this in terms of we are not making a sacrifice. We are doing what is necessary to keep the ecosystem that supports the human population.

Link to the video: [www.youtube.com/watch?v=NTbPNwNIoLs&ab_channel=UniversityofDelaware](http://www.youtube.com/watch?v=NTbPNwNIoLs&ab_channel=UniversityofDelaware).

Note: For the experiment, this video was shortened to 90 seconds by deleting the discussion about oak trees.
