# Supplementary material for: Encouraging pro-environmental behavior: Do testimonials by experts work?
Source: PLoS One. 2023 Oct 4;18(10):e0291612. doi: 10.1371/journal.pone.0291612 (PMC10550155; doi:10.1371/journal.pone.0291612)
Supplement: S2 Appendix — (DOCX) [file pone.0291612.s002.docx]

# S2 Appendix B. Experiment instructions and survey questions.
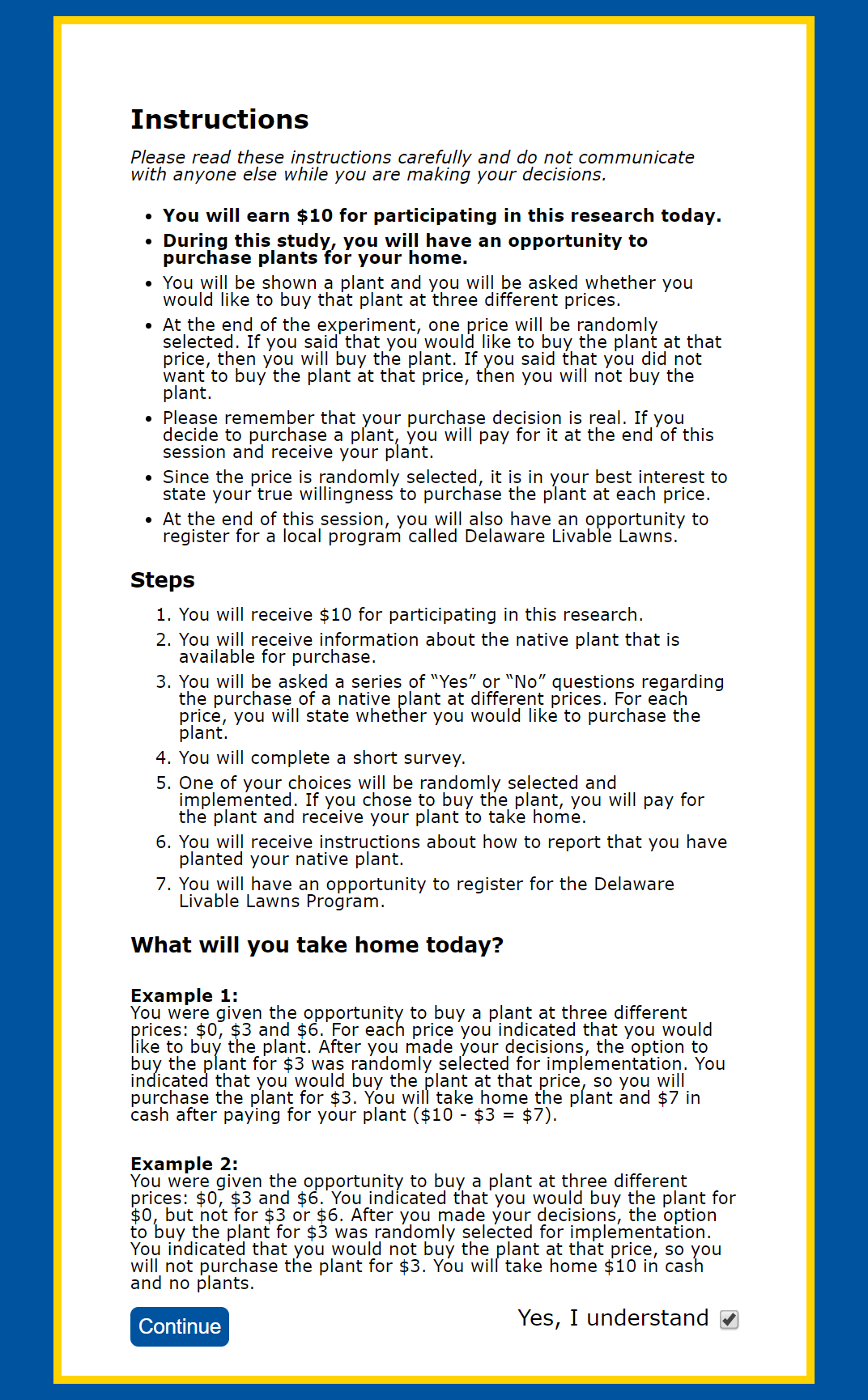


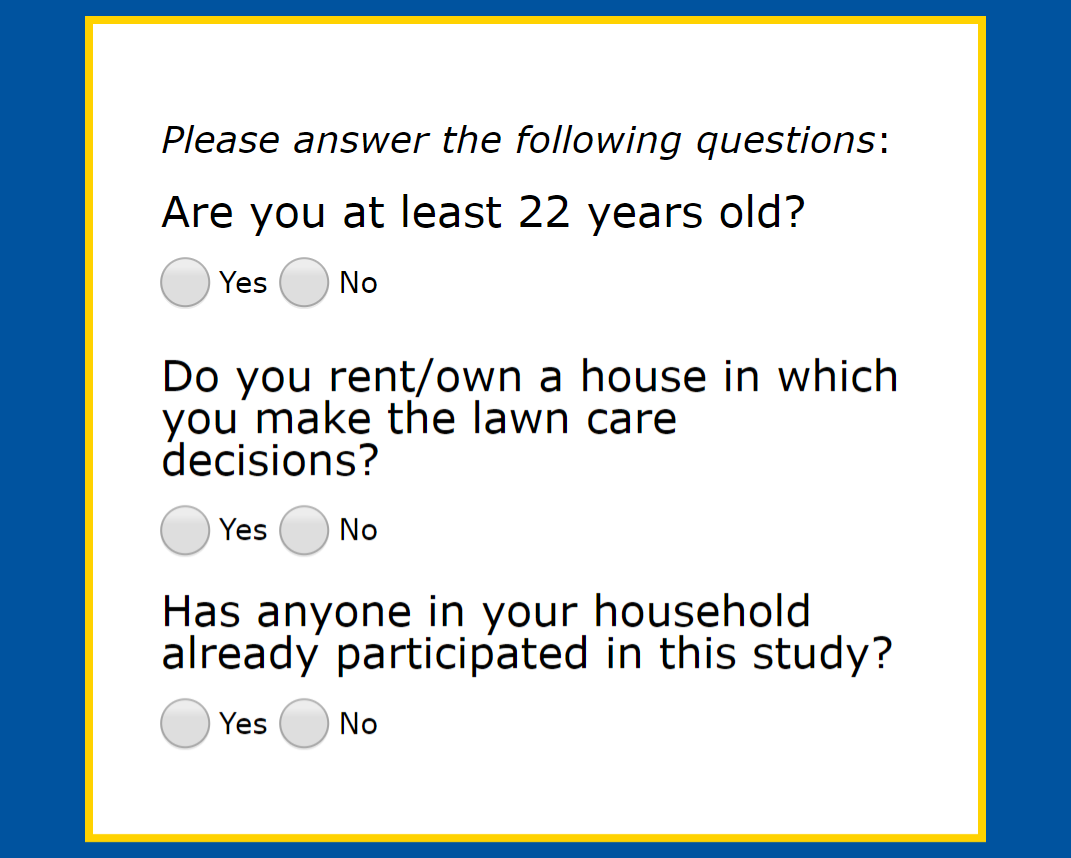


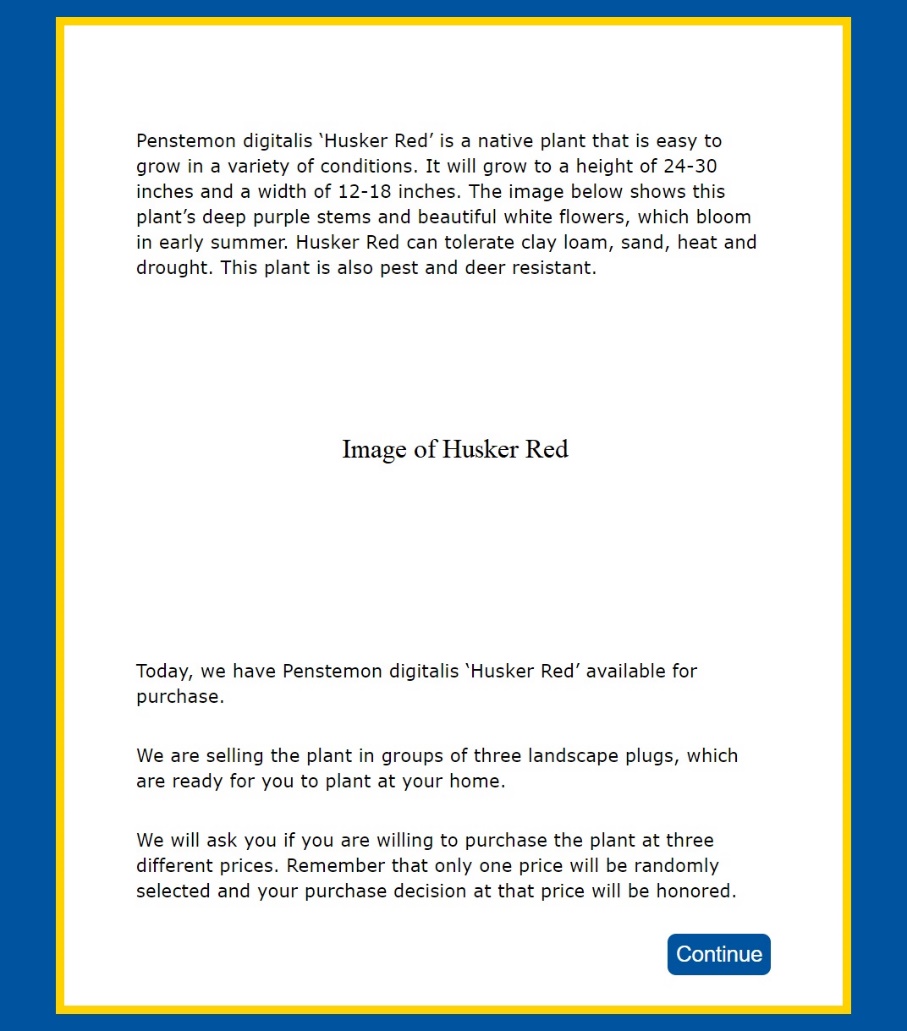


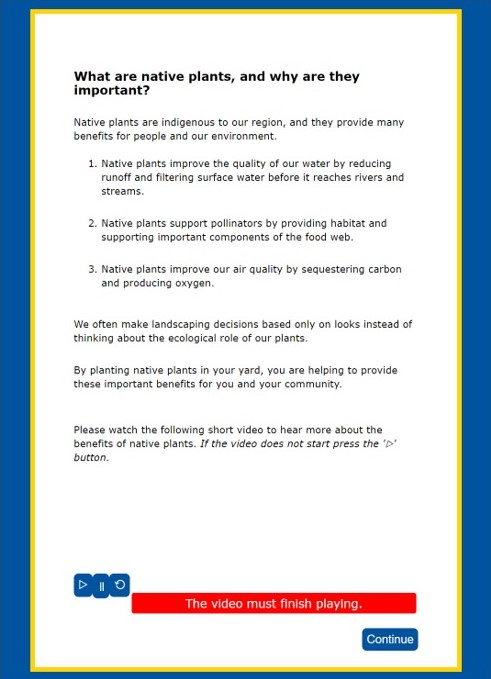


**Note:** Participants in the treatment saw this screen. Participants in the control group only saw the information preceding the video.


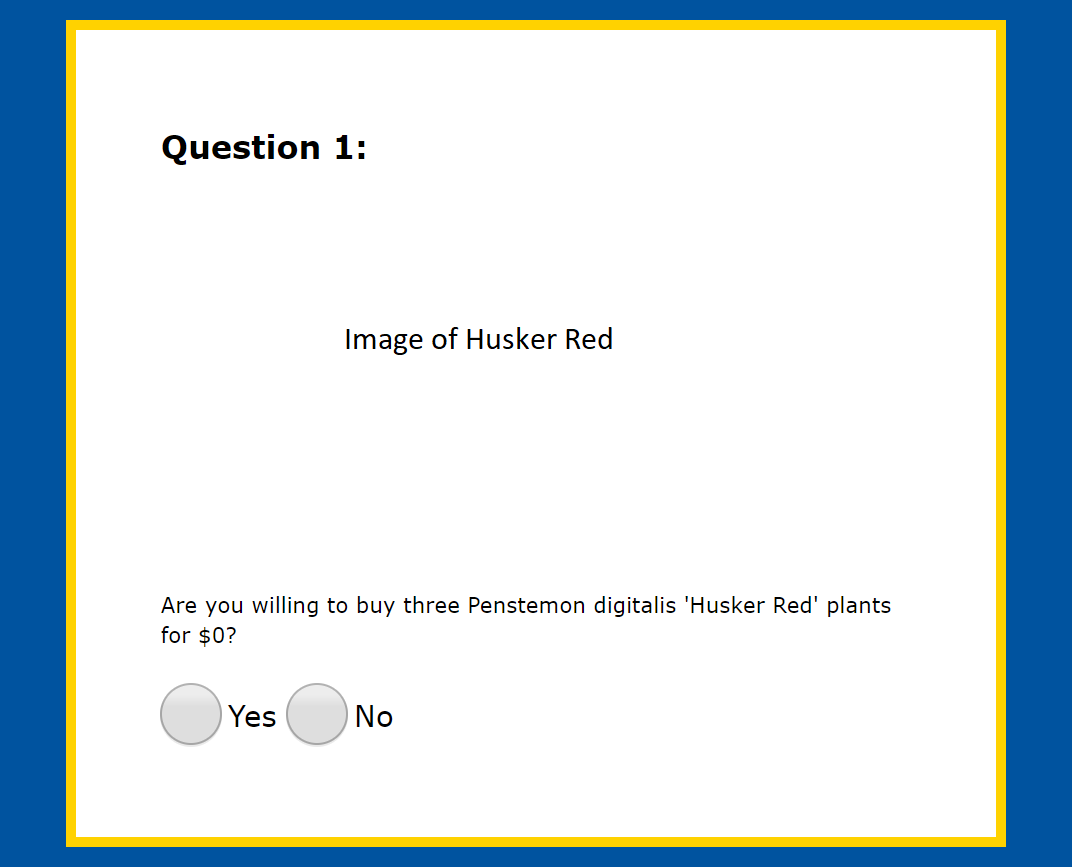


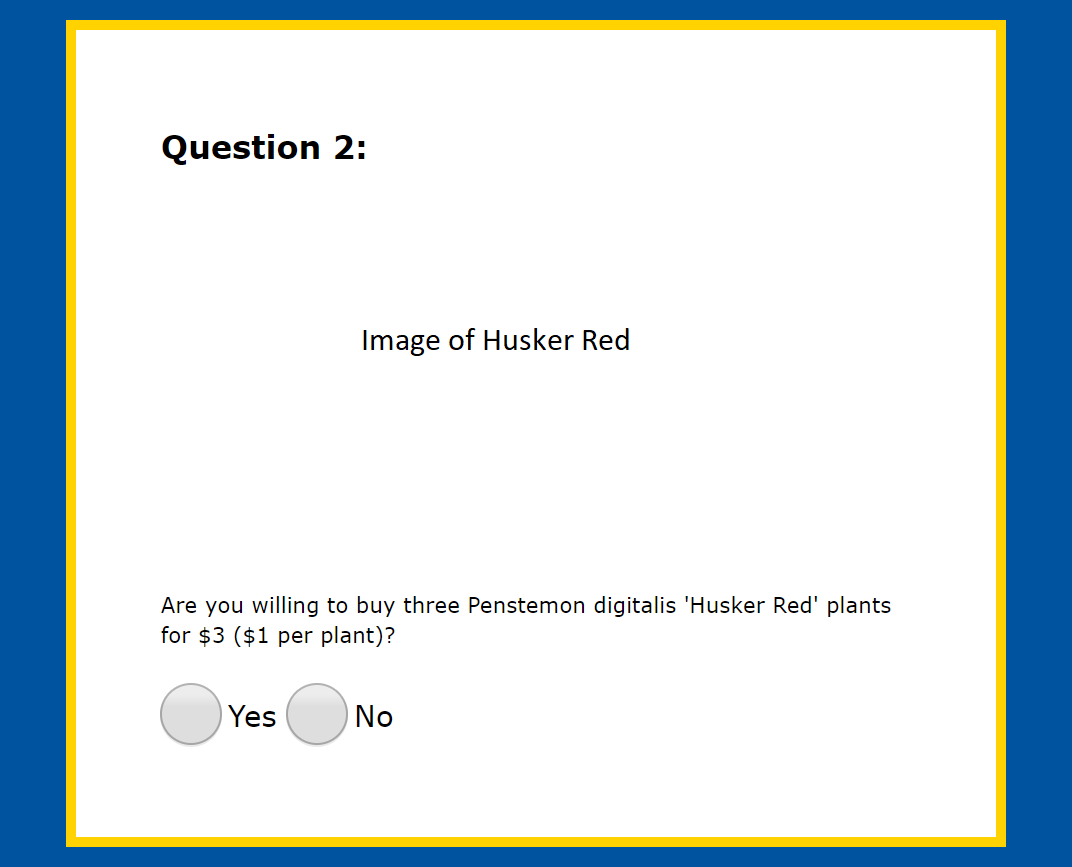


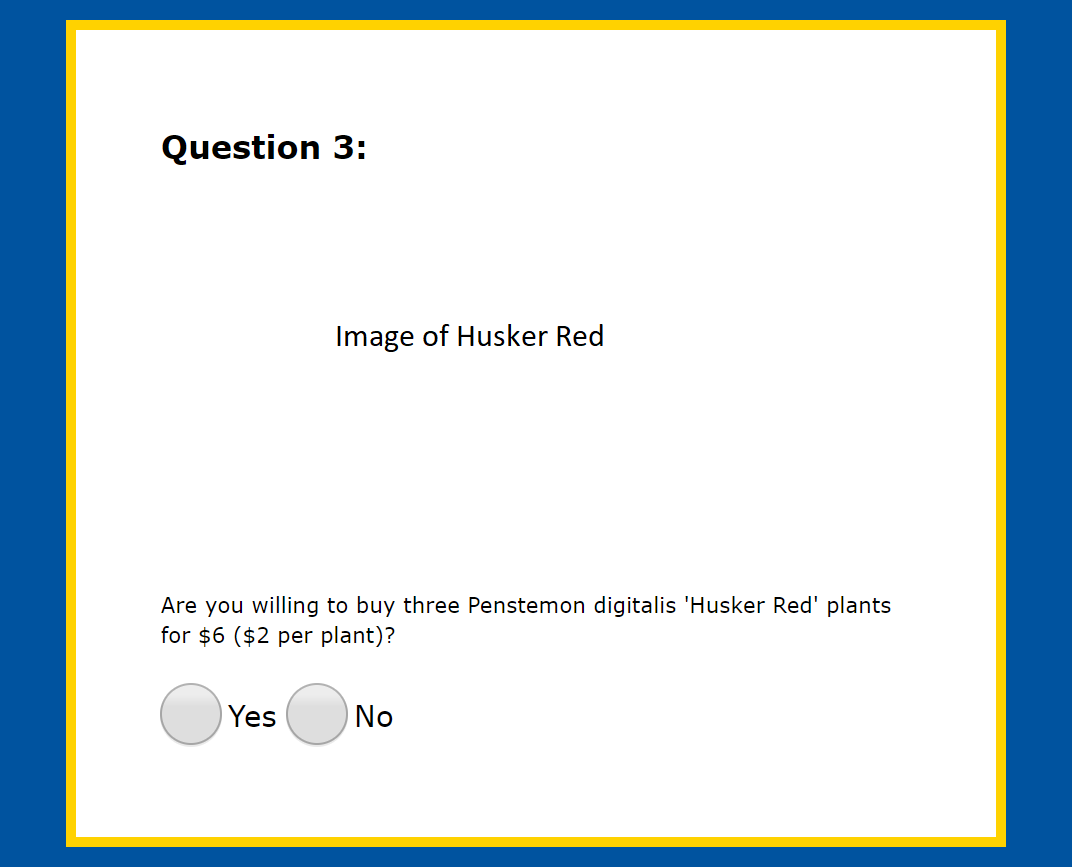


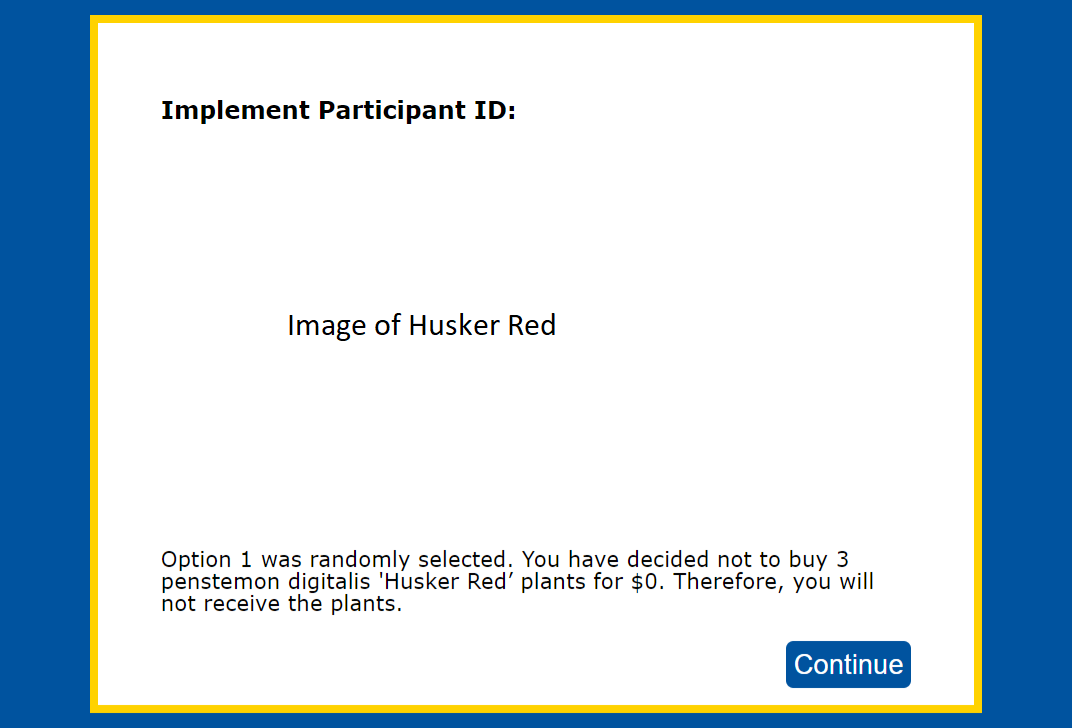


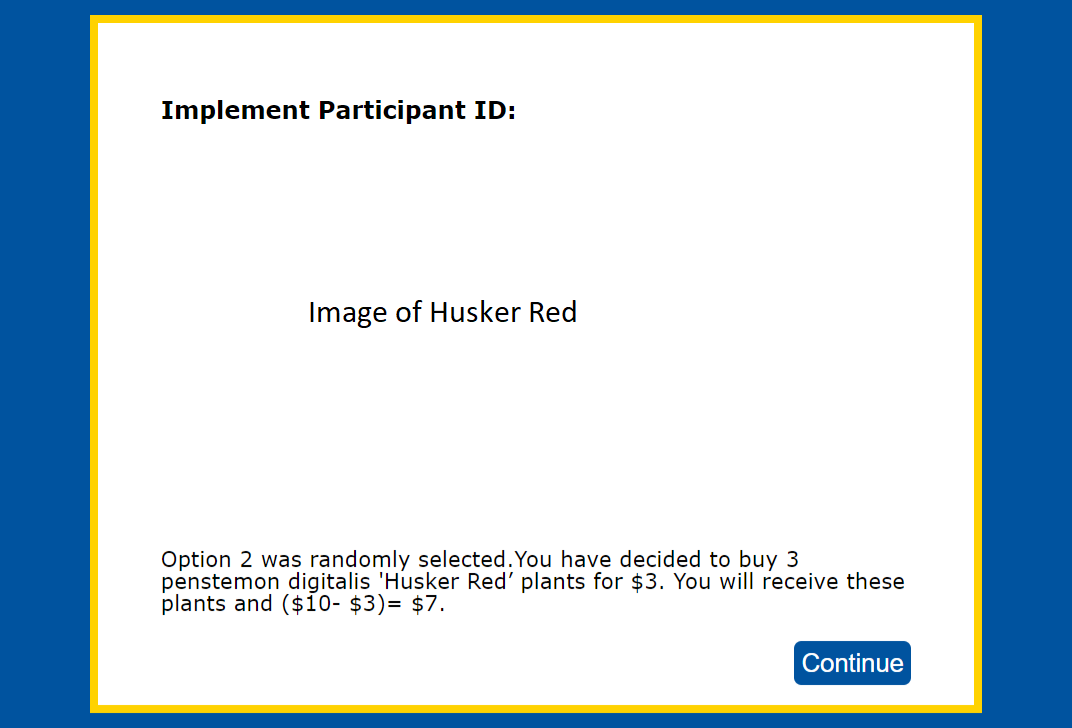


**
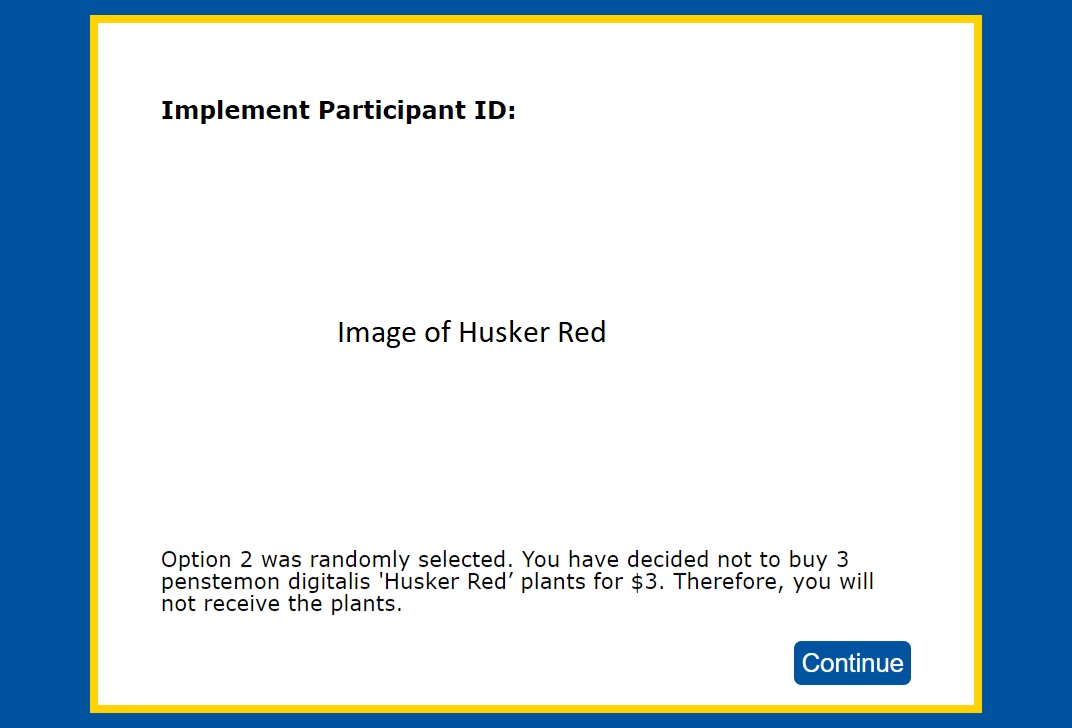
**

**
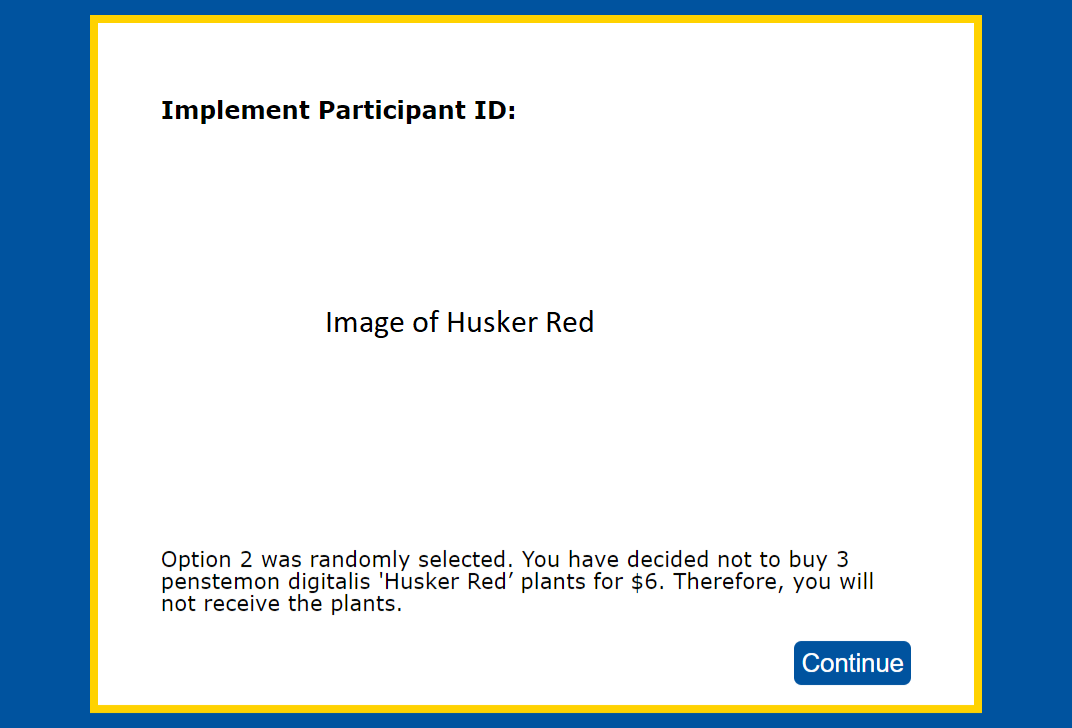
**

**
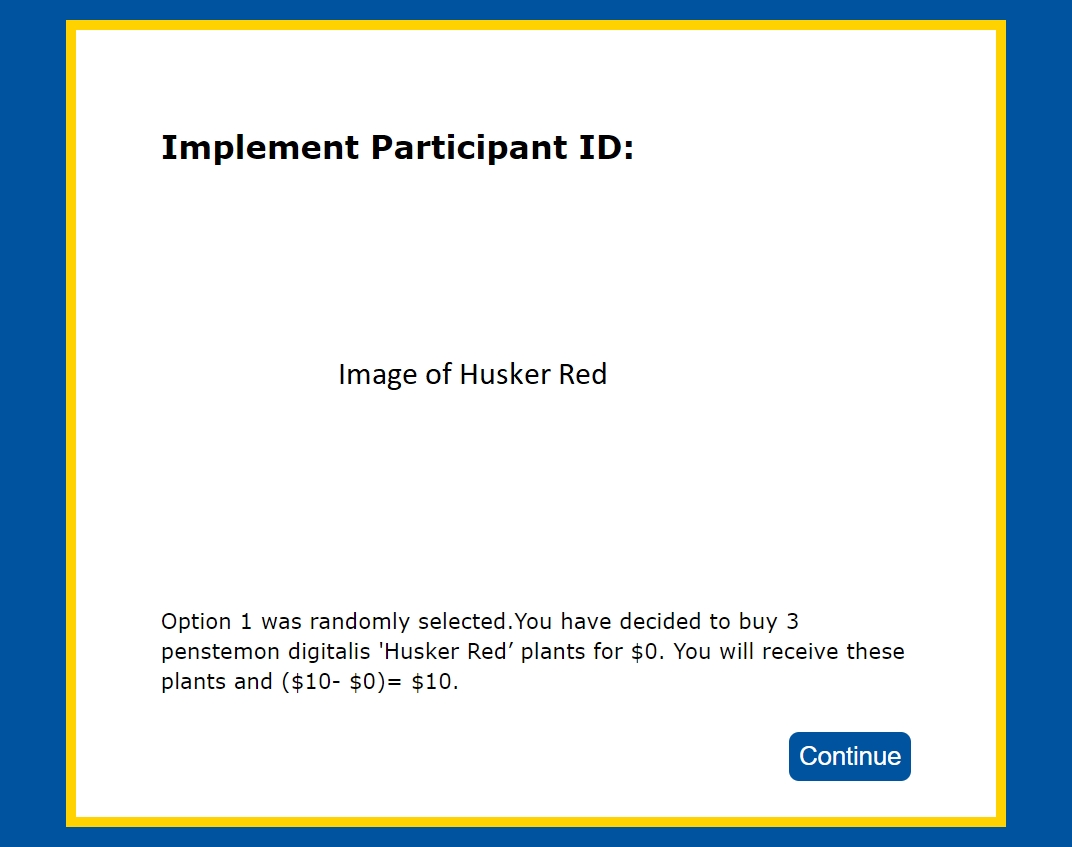
**

**
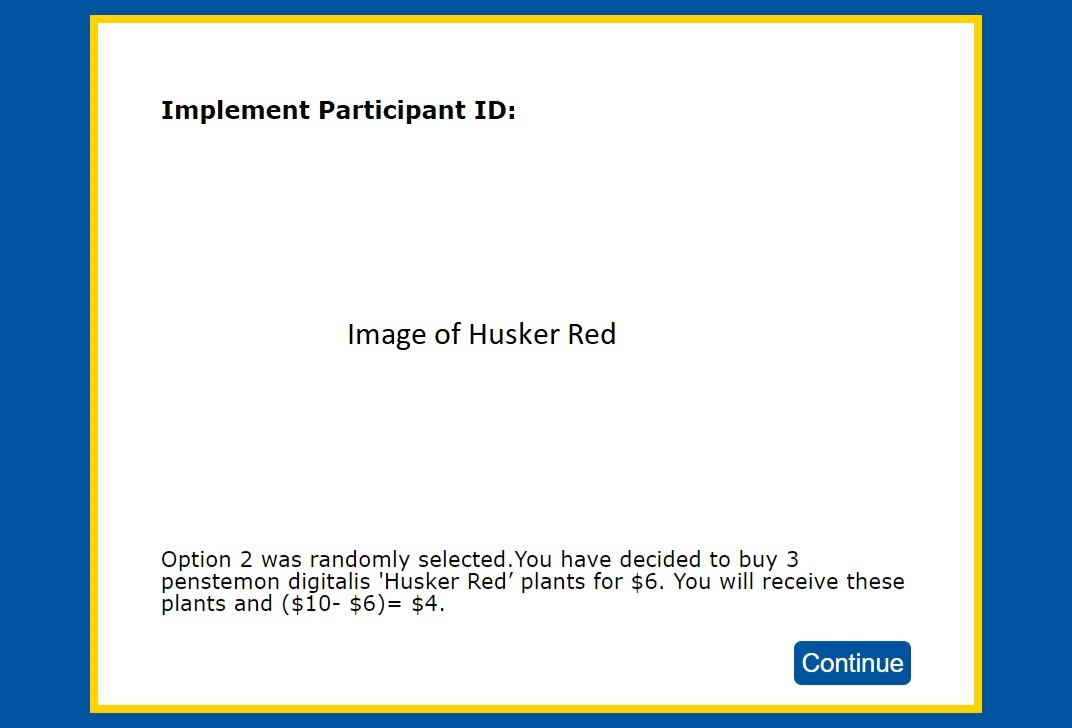
**

**Survey questions**

1. In what year were you born? (drop-down menu 1920–2000; can only participate if 1996 or before).
2. Who makes decisions about your lawn?

- I do
- Someone else in my household does
- My property manager/owner does
- Other (please specify) __________

1. What is your gender?

- Male
- Female
- Prefer not to answer

1. Which best describes the community that you live in?

- Rural
- Urban
- Suburban

1. Do you rent or own your own house?

- Rent
- Own

1. How many acres is your lawn? Please consider the area of your property that is comprised of grass, shrubs, and other plants. For comparison, a football field is approximately 1 acre.

- I do not have a lawn
- Less than ¼ acre
- ¼ – 0.99 acres
- 1––2 acres
- More than 2 acres

1. How many adults live in your household? __________
2. How many children (under 18 years old) live in your household? ______________
3. What is your ZIP code? __________[restrict to five digits]
4. Which best describes your employment status?

- Not employed
- Employed
- Retired
- Student
- Stay at home parent / caregiver

1. Are you:

- Politically liberal
- Politically moderate
- Politically conservative
- Other (please specify) __________

1. What best describes your ethnicity?

- White, not Hispanic
- Hispanic
- Black
- Asian
- Other (please specify) __________

1. Which category best describes your household income (before taxes) in 2017?

- Less than $25,000
- $25,000–$49,999
- $50,000–$74,999
- $75,000–$99,999
- $100,000–$149,999
- $150,000–$199,999
- $200,000 and above

1. What is the highest level of education that you have completed?

- Less than high school
- High school graduate or equivalent (i.e., GED)
- Some college, but no degree
- Associate degree
- Bachelor’s degree
- Graduate degree or professional degree

1. How concerned are you about the impact of your lawn care and landscaping decisions on the following factors?

|  | Not concerned | Somewhat concerned | Very concerned | Not sure |
| --- | --- | --- | --- | --- |
| Water quality |  |  |  |  |
| Biodiversity |  |  |  |  |
| Habitat for beneficial animals (ex: honey bees) |  |  |  |  |

1. Do you use any of the following environmentally-friendly practices at your home?

- Soil nutrient testing
- Follow fertilizer recommendations
- Water conservation practices (ex. soaker hoses, drip irrigation)
- Soil amendments (ex. biochar, peat moss)
- Native plants
- Rain barrels
- Other __________________

1. Have you heard about the Delaware Livable Lawns Program?

- Yes, but I am not a participant
- Yes, I have enrolled my lawn in the program
- No, I have not heard of it
