# Supplementary material for: Encouraging pro-environmental behavior: Do testimonials by experts work?
Source: PLoS One. 2023 Oct 4;18(10):e0291612. doi: 10.1371/journal.pone.0291612 (PMC10550155; doi:10.1371/journal.pone.0291612)
Supplement: Attachment — Submitted filename: PLOS One Review of PONE-D-22-27253-Encouraging pro-environmental behavior.docx [file pone.0291612.s003.docx]

**Review of ms PONE-D-22-27253: “Encouraging pro-environmental behavior: do testimonials by experts work?”**

**Reviewer’s summary**

A field experiment is reported in which 308 participants, visiting either a community event or a shopping mall or an ice cream shop, were asked to indicate their willingness to pay $ 0, 3 or 6 for an attractive native plant for their garden, which price – if they would purchase – was deducted from a sure $ 10 reward for their participation. Both a control group and a treatment group of each about 150 subjects first received written information about the nature of the experiment and what they were expected to do. In their instruction the planting of native plants was described as promoting biodiversity, preventing water runoff and sequestering carbon dioxide.

The treatment group was additionally presented with a 90-seconds’ video testimonial by a renowned plant-ecologist who essentially presented the same message in person. Main results indicate that there was no significant effect of the (additional) video presentation on subjects’ willingness to pay (at least about one dollar more than the control group) for the native plant offered. Besides there we some significant effects (e.g. of plant price, age, education) that could generally have been expected. The authors emphasize the importance of publishing negative effects of experimental treatments in the context of effectively encouraging pro-environmental behavior. And they conclude (a. o.) that “Our results make several important contributions.”

**General evaluation**

The paper is well written, the analysis seems appropriately done and the results are clearly reported. Given the nature and execution of the experiment, the importance of the results and their contribution to the professional literature seem somewhat inflated.

1. A fundamental problem in the design of the experiment is that all subjects were offered a $ 10 reward at the outset, and that their instruction implied that they would end up with the full $ 10 if they had expressed a zero willingness-to-pay for the bundle of three native plants or when they had expressed some WTP and were randomly assigned to the decision to purchase the plant for $ 0 (rather than $ 3 or 6). This may well have induced a bias for subjects to reduce any willingness to pay more than $ 0 for the plants.
2. Another problem is that the encouraging message about buying native plants was essentially the same for the control group and the treatment group, the latter differing only in a repeat and some extension of the message by the plant ecologist in person on video. So, was there really a substantive difference in treatment between the two groups?
3. A third problem may have resided in the unexpected invitation to participate in an experiment, while subjects had arrived with the intention (whatever) to attend the community event, to go shopping or to get an ice cream, respectively. It would seem that under such circumstances subjects being offered $ 10 for their (brief?) participation were naturally inclined to read instructions quickly and go along the presented questions soon enough to obtain their $ 10 reward, either or not lowered by the price of the bundle of plants they had purchased (or rather: had to purchase) following the study design, whereafter they would continue their journey in accordance with their original plan of visit.

**Some specific points**

a. The title of the paper is rather general. A more specific and informative version would be: ”Encouraging consumers to buy native plants: Do expert testimonials about ecological benefits work?”
b. The first sentence of the Abstract (and the first paragraph of the Introduction..) needs qualification. In many situations much depends on the various costs (whatever) for a person or household to adopt pro-environmental behavior, and upon other encouragement strategies such as product availability, relevant knowledge and abilities, and the like. [The authors cite Abrahamse’s (2019) book, rich in empirical information about ‘what works, what doesn’t and why’.] The *last* sentence of the abstract provides non-surprising information which is unrelated to the difference between experimental conditions.
c. On p. 5, line 109, a phrase like “.. an incentive-compatible single-bounded dichotomous-choice framed field experiment” sounds unnecessarily complicated. Readers might want to know what ‘incentive-compatible’ and ‘single-bounded’ practically mean. And ‘binary’ seems simpler than ‘dichotomous’.
d. Note that data collection for the reported experiment was in 2018, *i.e.* at least four years ago. Why such delay?
e. On p. 6, lines 122-123, sentence “Participants completed the experiment asynchronously and were not permitted to communicate with other participants present at the same time” raises questions about the practical setting in which subjects had to perform the requested task. See also general problem 3 above.
f. Also on p. 6, lines 141-142: the three-point WTP-scale of $ 0, 3 and 6 may not have been sensitive or extensive enough to assess the possible added effects of the video-message following the written instruction.
g. A nice aspect of the paper is the authors’ exercise about the (in-) sufficiency of statistical power to detect possible treatment effects. On p. 15, lines 284-285, the authors conclude that “Based on our power analyses, we cannot rule out the possibility that the testimonial has [or rather: ‘had’?] an effect below the $0.96 to $1.50 range.” If so, this might have meant that subjects were willing to pay (approximately) fifty cents more in response to the video-message. Would the authors have found this an interesting result worth publishing in a professional journal? [And note again general problem 2 above.]
h. Experienced researchers in ‘encouraging pro-environmental behavior‘ might object to the strong generalization speaking from the passage on p. 16, lines 307-310: “Contrary to studies of testimonials used in other contexts […], we find that the expert’s video testimonial had no significant identifiable effect on WTP for native plants. Consequently, in pro-environmental contexts, testimonial-based interventions alone are not necessarily sufficient to motivate changes in behavior.” The generality of this conclusion does not seem entirely warranted.
