# Supplementary material for: Encouraging pro-environmental behavior: Do testimonials by experts work?
Source: PLoS One. 2023 Oct 4;18(10):e0291612. doi: 10.1371/journal.pone.0291612 (PMC10550155; doi:10.1371/journal.pone.0291612)
Supplement: Attachment — Submitted filename: Response to Reviewers.docx [file pone.0291612.s004.docx]

Author’s response to comments by Reviewer #1: REVIEW OF PONE-D-22-27253

Title: "Encouraging pro-environmental behavior: Do testimonials by experts work"

Thank you for reading our manuscript and for the helpful comments and suggestions. By responding to your comments and the comments of the other referees, we believe our manuscript has significantly improved. You will find your original comments in **bold**, our response to your comments immediately afterwards, and then the sections of revised text, as appropriate.

**In their research, the authors tested the effectiveness of an expert’s video testimonial in promoting people’s willingness to pay for native plants. In their field experiment with 308 volunteers, the authors did not find the expected effect of the testimonial. The authors nevertheless believe that, in addition to reports of successful behavior-change interventions, reports of negative results (i.e., indicating ineffective interventions) are also worth knowing about.

The paper is well-written and conventionally organized. The research objective is explicitly stated (see p. 4), and the experimental trial is properly conducted and reported. On the negative side, I find the focus in the Introduction on nudging misleading, as nudges are not only non-monetary behavioral interventions. The discussion is scant and should address some more alternative explanations for why the intervention could have failed. Moreover, some of the technicalities are not spelled out in enough detail. Although ineffective interventions leave much room for speculation on why they failed, this research nevertheless offers a valuable contribution to the literature on the promotion of pro-environmental behavior. In the following, I will elaborate on my appraisal in more detail.**

1. **Nudges are not only non-monetary influences on behavior. Rather, the term actually refers to aspects of the “choice architecture” that either facilitate the target behavior or impede the behavioral alternative (see, e.g., Taube & Vetter, 2019). Such facilitating/impeding structural factors are supposed to modify the boundary conditions in which a behavior or a decision takes place. They can and should be separated from providing information in all its forms (e.g., instructing, advertising, and messaging; see Osbaldiston & Schott, 2012). Whereas facilitating factors originate from rearranging the conditions in which a behavior takes place (e.g., by making something more accessible), the testimonial in this study provides sound reasons for some specific gardening practices (i.e., why information).**

Thank you for this helpful comment. We edited the language in the abstract and in the manuscript to remove references to nudges and make it clear that we are focusing on non-monetary behavioral interventions and expert testimonials, specifically.

Abstract: “Using non-pecuniary interventions to motivate pro-environmental behavior appeals to program administrators seeking cost-effective ways to increase adoption of environmental practices. However, we should not expect that all interventions are effective and reporting when interventions fail is as important as documenting their successes. We used a framed field experiment with 308 adults from the Mid-Atlantic in the United States to test the effectiveness of an expert testimonial in encouraging adoption of native plants in residential settings. Though studies have found testimonials to be effective in other contexts, we find that the video testimonial had no effect on residents’ willingness to pay for native plants. Our analysis also shows that consumers who are younger, have higher incomes, and use other environmentally friendly practices on their lawns are more likely than other consumers to purchase native plants.”

[Page 2]: “Policymakers and program managers are increasingly interested in identifying effective interventions to promote pro-environmental behavior [1, 2]. Growing evidence suggests that non-pecuniary behavioral interventions have the potential to motivate energy and water conservation [3–8], lead to more environmentally friendly food choices [9], improve recycling efforts [10], and reduce nonpoint source pollution [11]. However, gaps remain in our understanding of the effectiveness of specific interventions in motivating pro-environmental behavior [12, 13]. Based on a review of 160 experiment-based studies of environmental interventions, Byerly et al. [12] identified areas in need of future research to inform the design of policies and programs, including testing the effectiveness of interventions used in other contexts in promoting pro-environmental behavior. Behavioral interventions such as information provision [14, 15], peer comparisons [16], social norms [17,18], and framing [19,20] have been extensively studied. Less is known about testimonials such as opinions and recommendations provided by experts and influencers who speak positively about a pro-environmental product or practice.”

2. **Another issue worth considering is the specific pro-environmental behavior under investigation: the procuring of a specific native plant. Are its “costs” a possible explanation for its relative lack of malleability (see, e.g., Kaiser et al., 2021). If the behavior is obstructed by costs (e.g., plant care effort and time demands) or the availability of an area that is ready for landscaping, only people with a comparatively strong commitment to environmental protection (i.e., with strong environmental attitudes) would respond favorably to the testimonial. Without knowledge about people’s attitudes and the relative costs of this particular behavior, we cannot rule out the challenging nature of gardening in general or the insufficient environmental attitude of the experimental group as alternative explanations of the intervention’s inefficacy (see Kaiser et al., 2010).**

Thank you for pointing this out. We included your suggestion as a potential explanation for the treatment’s ineffectiveness as follows:

[Page 15]: “There may be several potential reasons that could explain why the expert testimonial treatment had no effect on participants’ WTP to purchase native plants. First, transaction costs associated with planting (e.g., finding or clearing the area where to plant, time and effort required to plant and care for the plants, etc.) could be perceived as high by some participants. Transaction costs have been shown to be a barrier to adoption of pro-environmental behavior in other contexts, such as adoption of agricultural conservation practices [57,58] and residential landscape best management practices [59], among others. Further, without information about the level of the participants’ commitment to environmental protection, it is unclear whether the transaction costs or insufficient strength of environmental attitudes to offset these costs [60,61] may have led to the ineffectiveness of the testimonial treatment.”

3. **The differences between the treatment and control groups speak of not fully comparable subsamples and a somewhat failed random allocation (see Table 1). These different subsample characteristics could have biased the findings of this research. In their Model 2, the authors statistically controlled for such confounding influences (see Table 2). Nevertheless, there might still be other—unidentified—confounding factors (e.g., group differences in environmental attitude) that could have masked the efficacy of the testimonial.**

We appreciate your concern and have included a discussion of this issue in the revised manuscript as follows:

[Page 16]: “Second, while we used randomized assignment to treatments and controlled for the characteristics that were not balanced across the treatment and control groups in our analysis, other unidentified confounding factors (e.g., group differences in environmental attitudes) may have potentially precluded the identification of a statistically significant treatment effect.”

4. **Table 1 additionally speaks of some nonresponse error. People who volunteered for this research differed from people who did not volunteer, a difference that can be seen in the sample’s remarkable departure from the population in Delaware in educational level. The much higher education level of the sample could alternatively explain the inefficacy of the testimonial. Due to their advanced educational levels, participants might already possess the knowledge conveyed in the testimonial, which could account for the inefficacy of the testimonial.**

Good point. We included this potential driver of the inefficacy of the testimonial in the paper as follows:

[Page 16]: “Finally, the participants in our experiment tended to be more educated compared to the general population in Delaware and could have already possessed some of the knowledge communicated in the testimonial, which could have led to the lack of a significant impact of the testimonial on participants’ preferences.”

**In the following, I list some more issues that also need amendment before the paper will be ready for publication:**

5. **Please specify the effect size measure (d, g, r) that you report (e.g., p. 8).**

We implemented our simulation to determine the sample size required to detect a 0.3 standard deviation difference in the mean WTP for native plants between our treatment (testimonial video) and control groups; i.e., we used a d family effect size measure of d=0.3. We have revised the text to read:

[Page 8]: “The final experimental design required 345 participants to reliably detect the effect of the video-testimonial on mean WTP for native plants between the treatment and control groups. To conduct the simulation, we assumed a standardized effect size of d=0.30 with 80% power (i.e., $\beta=0.80)$.”

6. **What algorithm was used for the random presentation of prices (p. 7)?**

The experiment platform was developed using Willow, a Python framework for programming economic experiments (Weel and McCabe 2009). Random selection of the price treatments was implemented within the experiment platform. This is noted on page 6:

“Random assignment of participants to the control and testimonial treatments was implemented within the experiment platform that was developed using Willow, a Python framework for programming economic experiments [51]. Willow also did the random selection of which of the price treatments.”

51. Weel, J., McCabe, K., 2009. Willow: Experiments in Python. George Mason University. http://econwillow.sourceforge.net

7. **Also, specify the randomization procedure that was applied to randomly allocate participants to the experimental and control groups (p. 6). Doing so might help readers better understand the suboptimal allocation to groups (see Table 1).**

When participants started the experiment, they were assigned to either the treatment or control group. Of the 308 participants, 154 were in each group. The experiment platform was developed using Willow, a Python framework for programming economic experiments (Weel and McCabe 2009). As noted above, the random assignment to treatments was implemented within the experiment platform.

8. **Provide the results of the t tests in more detail. Explain the “joint orthogonality tests,” their purpose, and your interpretation (p. 9).**

Thank you for this suggestion. We have added a new column in Table 1. The new column shows the difference between control and treatment groups with p values of t-tests in parentheses.

In addition, we conducted a test of joint orthogonality. The joint orthogonality test is complementary to t-tests on each individual variable. The purpose of a joint test of orthogonality is to check whether all socio-demographic variables are balanced (i.e., statistically indifferent) between the control and treatment groups. We first ran a logit regression where the dependent variable is the treatment dummy variable, and the independent variables are socio-demographic variables. With the regression results (say coefficient estimates are b1, b2, b3, …), we tested the joint hypothesis (b1=b2=b3=…b10=0). Since we rejected the null hypothesis (at a 1% significance level), we concluded that the control and treatment groups are not balanced across all socio-demographic variables.

Please find the edits we made to the paper and the updated table 1 below.

[Page 10]: “In addition, we conducted a test of joint orthogonality. The joint orthogonality test is complementary to t-tests on each individual variable. The purpose of a joint test of orthogonality is to check whether all socio-demographic variables are balanced (i.e., statistically indifferent) between the control and treatment groups. The results of the joint orthogonality test showed that the control and treatment groups are not balanced across all socio-demographic variables.”

[Page 9]: **Table 1. Summary statistics for socio-demographic variables by treatment.**

| **Variable** | **Definition** | **Entire Sample** | **Control** | **Treatment** | **Difference (Control-Treatment)** | **Delaware (2018)**^a^ |
| --- | --- | --- | --- | --- | --- | --- |
| Age | Participant’s age in years in 2018 | **40.70** | **41.12** | **40.28** | **0.844** | **40.20** |
|  |  | (15.00) | (15.35) | (14.65) | (0.393) | (Median) |
| Male | Equals 1 for male participants | **0.438** | **0.494** | **0.383** | **0.110^***^** | **0.484** |
|  |  | (0.496) | (0.500) | (0.487) | (0.001) |  |
| Children | Equals 1 when a child under 18 resided in the household | **0.416** | **0.370** | **0.461** | **-0.091^***^** | **0.252** |
|  |  | (0.493) | (0.483) | (0.499) | (0.005) |  |
| Employed | Equals 1 when participant was employed | **0.659** | **0.662** | **0.656** | **0.006** | **0.586** |
|  |  | (0.474) | (0.473) | (0.476) | (0.835) |  |
| Income_<$50K_ | Equals 1 when participant’s income was less than $50,000 | **0.315** | **0.344** | **0.286** | **0.058**^*^ | **0.383** |
|  |  | (0.465) | (0.476) | (0.452) | (0.056) |  |
| Income_$50K-$100K_ | Equals 1 when participant’s income was between $50,000 and $100,000 | **0.354** | **0.299** | **0.409** | **-0.110^***^** | **0.322** |
|  |  | (0.478) | (0.458) | (0.492) | (0.000) |  |
| Income_>$100K_ | Equals 1 when participant’s income was greater than $100,000 | **0.331** | **0.357** | **0.305** | **0.052** | **0.296** |
|  |  | (0.471) | (0.480) | (0.461) | (0.094) |  |
| Education level | Equals 1 for participants who had a bachelor, graduate, or professional degree | **0.672** | **0.682** | **0.662** | **0.019** | **0.314** |
|  |  | (0.470) | (0.466) | (0.473) | (0.529) |  |
| Urban | Equals 1 for participants living in urban areas | **0.873** | **0.890** | **0.857** | **0.032** |  |
|  |  | (0.333) | (0.314) | (0.350) | (0.138) |  |
| Homeowner | Equals 1 for participants who owned their homes | **0.679** | **0.662** | **0.695** | **-0.032** |  |
|  |  | (0.467) | (0.473) | (0.461) | (0.291) |  |
| Lawnsize_small_ | Equals 1 when lawn size was less than 0.25 acres or participants did not have a lawn | **0.425** | **0.422** | **0.429** | **-0.006** |  |
|  |  | (0.495) | (0.494) | (0.495) | (0.842) |  |
| Lawnsize_medium_ | Equals 1 when lawn size was between 0.25 and 1.0 acres | **0.448** | **0.435** | **0.461** | **-0.026** |  |
|  |  | (0.498) | (0.496) | (0.499) | (0.428) |  |
| Lawnsize_large_ | Equals 1 when lawn size was greater than 1.0 acres | **0.127** | **0.143** | **0.110** | **0.032** |  |
|  |  | (0.333) | (0.350) | (0.314) | (0.138) |  |
| Lawn practice | Equals 1 when participants use environmentally friendly practices at home for the lawn | **0.844** | **0.831** | **0.857** | **-0.026** |  |
|  |  | (0.365) | (0.375) | (0.350) | (0.277) |  |
| **N** |  | **308** | **154** | **154** |  | **949,495** |

In the first three columns, mean values are bolded. Standard deviations are in parentheses. The column labeled “Difference (Control-Treatment)” has mean values in bold and p values of t-tests in parentheses. ***, **, and * denote statistical significance at the 1%, 5%, and 10% level respectively. ^a^Source: 2018 American Community Survey: www.census.gov/acs/www/data/data-tables-and-tools/data-profiles/2018/.

9. **For completeness sake, in addition to the main effects of price and testimonial, I would also like to see the interaction between price and testimonial included in the random effects logistic regression model as an additional effect (p. 12).**

Thank you for this suggestion. We have added an interaction term between price and testimonial to the random effect logistic regression model, presented as Model 3 in Table 2.

[Page 12]: “We present the results for the simple model that estimated only the treatment effect (Model 1), the expanded model that included the full set of socio-demographic characteristics (Model 2), and the expanded model with an interaction term between price and testimonial (Model 3). Models 2 and 3 produced similar results.”

[Page 13]: **Table 2. Random effects logistic regression results.**

|  | **Model 1** | | **Model 2** | | **Model 3** | |
| --- | --- | --- | --- | --- | --- | --- |
|  | **Coeff.** | **S.E.** | **Coeff.** | **S.E.** | **Coeff.** | **S.E.** |
| Price | -0.654^***^ | (0.067) | -0.654^***^ | (0.067) | -0.648^***^ | (0.081) |
| Testimonial | 0.161 | (0.306) | 0.111 | (0.307) | 0.148 | (0.390) |
| Price*Testimonial |  |  |  |  | -0.012 | (0.093) |
| Age |  |  | -0.038^***^ | (0.012) | -0.038^***^ | (0.012) |
| Male |  |  | 0.160 | (0.307) | 0.160 | (0.306) |
| Children |  |  | -0.300 | (0.317) | -0.299 | (0.317) |
| Employed |  |  | -0.230 | (0.320) | -0.232 | (0.320) |
| Income_$50K-$100K_ |  |  | 0.933^**^ | (0.403) | 0.933^**^ | (0.404) |
| Income_>$100K_ |  |  | 0.818^*^ | (0.448) | 0.819^*^ | (0.448) |
| Urban |  |  | -0.216 | (0.558) | -0.217 | (0.559) |
| Education level |  |  | 0.304 | (0.333) | 0.305 | (0.333) |
| Homeowner |  |  | 0.525 | (0.362) | 0.524 | (0.362) |
| Lawnsize_medium_ |  |  | -0.099 | (0.342) | -0.098 | (0.342) |
| Lawnsize_large_ |  |  | 0.790 | (0.506) | 0.788 | (0.506) |
| Lawn practice |  |  | 0.764^*^ | (0.409) | 0.764^*^ | (0.408) |
| Constant | 1.983^***^ | (0.301) | 2.070^**^ | (0.808) | 2.051^**^ | (0.805) |
|  |  | |  |  |  |  |
| Log pseudolikelihood | -504.192 | | -491.869 | | -491.858 | |
| N | 924 | | 924 | | 924 | |
| ^***^, ^**^, and ^*^ denote statistical significance at the 1%, 5%, and 10% level respectively. | | | | | | |

10. **The text ends in a rather dull manner with the limitations of the research (p. 17). The paper should end with a major conclusion rather than with what Bem (1987) calls a “whimper.”**

We appreciate this suggestion. We revised the manuscript so that it (hopefully) does not end with a “whimper”, but instead with a compelling summary of our findings. We provided suggestions for future research in the Results section:

[Page 16]: “Future studies can extend this research by testing whether any of the factors described above could have an impact on the effectiveness of expert testimonials on pro-environmental behavior of buying plants. To further inform policymakers and program administrators, future studies can also test the efficacy of testimonial-based interventions in the context of other pro-environmental behavior, examine the effect of combining testimonials with other non-monetary behavioral interventions and/or financial incentives, or using larger samples to detect smaller effects of testimonials alone.”

[Page 18]: “Our results make several important contributions. First, we extend the literature examining non-monetary interventions that rely on information provision on adoption of pro-environmental behavior by analyzing the effect of expert testimonials using a carefully designed, revealed preference experiment. Publishing null results and presenting results from power analyses are critical in supporting efforts to address publication bias that occurs when only statistically significant findings are reported [63]. Finally, our findings have practical implications, particularly for environmental organizations and government agencies considering using expert testimonials to increase engagement in their programs. Our results show that such efforts should consider whether the cost of creating and disseminating testimonials is worth the expected benefit, which could be small or non-existent based on the study’s results.”

11. **In conclusion, this research seems to be by and large competently conducted and reported. And despite the failed intervention, I recommend that this paper be published after it is revised, as it can make a valuable contribution to environmental protection research.**

Thank you for this comment. We appreciate your valuable suggestions and believe that they have significantly improved our manuscript. We agree with you that publishing interventions that fail is just as important as documenting those that are successful.

REFERENCES

Bem, D. J. (1987). Writing the empirical journal article. In M. P. Zanna & J. M. Darley (Eds.), The compleat academic: A practical guide for the beginning social scientist (pp. 171-201). McGraw-Hill.

Kaiser, F. G., Byrka, K., & Hartig, T. (2010). Reviving Campbell's paradigm for attitude research. Personality and Social Psychology Review, 14, 351-367.

Kaiser, F. G., Kibbe, A., & Hentschke, L. (2021). Offsetting behavioral costs with personal attitude: A slightly more complex view of the attitude-behavior relation. Personality and Individual Differences, 183, 111158.

Taube, O., & Vetter, M. (2019). How green defaults promote environmentally friendly decisions: Attitude‐conditional default acceptance but attitude‐unconditional effects on actual choices. Journal of Applied Social Psychology, 49, 721–732.

Osbaldiston, R., & Schott, J. P. (2012). Environmental sustainability and behavioral science: Meta-analysis of proenvironmental behavior experiments. Environment and Behavior, 44, 257–299.

Author’s response to comments by Reviewer #2: REVIEW OF PONE-D-22-27253

Title: "Encouraging pro-environmental behavior: Do testimonials by experts work"

Thank you for reading our manuscript and for your comments. You will find your original comments in **bold**, our response to your comments immediately afterwards, and then the sections of revised text, as appropriate.

**Reviewer’s summary**

**A field experiment is reported in which 308 participants, visiting either a community event or a shopping mall or an ice cream shop, were asked to indicate their willingness to pay $ 0, 3 or 6 for an attractive native plant for their garden, which price – if they would purchase – was deducted from a sure $ 10 reward for their participation. Both a control group and a treatment group of each about 150 subjects first received written information about the nature of the experiment and what they were expected to do. In their instruction the planting of native plants was described as promoting biodiversity, preventing water runoff and sequestering carbon dioxide.**

**The treatment group was additionally presented with a 90-seconds’ video testimonial by a renowned plant-ecologist who essentially presented the same message in person. Main results indicate that there was no significant effect of the (additional) video presentation on subjects’ willingness to pay (at least about one dollar more than the control group) for the native plant offered. Besides there we some significant effects (e.g. of plant price, age, education) that could generally have been expected. The authors emphasize the importance of publishing negative effects of experimental treatments in the context of effectively encouraging pro-environmental behavior. And they conclude (a. o.) that “Our results make several important** **contributions.”**

**General evaluation**

**The paper is well written, the analysis seems appropriately done and the results are clearly** **reported. Given the nature and execution of the experiment, the importance of the results and their contribution to the professional literature seem somewhat inflated.**

1. **A fundamental problem in the design of the experiment is that all subjects were offered a $10 reward at the outset, and that their instruction implied that they would end up with the full $ 10 if they had expressed a zero willingness-to-pay for the bundle of three native plants or when they had expressed some WTP and were randomly assigned to the decision to purchase the plant for $ 0 (rather than $ 3 or 6). This may well have induced a bias for subjects to reduce any willingness to pay more than $ 0 for the plants.**

We appreciate your concerns, however, we restfully disagree that this is a fundamental problem. In fact, providing compensation to subjects for participating in economic experiments is a widely accepted practice and a critical component of conducting experimental research that allows researchers to model behavior in non-hypothetical settings (Voslinsky and Azar, 2021, Davis et al. 2010). Research also suggests that providing compensation increases participation (Krawczyk, 2011, Abeler and Nosenzo, 2015; Gajic et al, 2012), especially in contexts when getting subjects to participate may be challenging (Kerr et al., 2012) and this can help reduce bias that may arise from having only voluntary participants.

In sum, we followed well-established practices in experimental economics and previous literature to design our experiment. Subjects in our experiment were endowed with $10 at the beginning of the experiment that they could spend on purchasing plants, if they wanted. While more participants were willing to get the plants for free, a substantial number of participants were willing to pay $3 or $6 (see Fig. 3). Following common protocols in experimental economics, participants were told that only one of the three purchase decisions would be binding, and that the binding decision would be randomly selected at the end of the study to determine participants’ take-home cash and whether they purchased the native plants. This mechanism ensured that participants had no incentive to misrepresent their WTP as their dominant strategy was to answer ‘Yes’ whenever the posted price did not exceed their maximum WTP for the bundle. In addition, since participants in both control and treatment groups received the same $10 as compensation, this compensation unlikely affected our ability to examine the impacts of testimonial video on participants’ willingness to pay, which was a key question of this research.

References

Abeler J, Nosenzo D. Self-selection into laboratory experiments: pro-social motives versus monetary incentives. Experimental Economics. 2015 Jun;18:195-214.

Canavari M, Drichoutis AC, Lusk JL, Nayga Jr RM. How to run an experimental auction: A review of recent advances. European Review of Agricultural Economics. 2019 Dec 1;46(5):862-922.

Davis LR, Joyce BP, Roelofs MR. My money or yours: house money payment effects. Experimental Economics. 2010 Jun;13:189-205.

Gajic A, Cameron D, Hurley J. The cost-effectiveness of cash versus lottery incentives for a web-based, stated-preference community survey. The European Journal of Health Economics. 2012 Dec;13:789-99.

Kerr J, Vardhan M, Jindal R. Prosocial behavior and incentives: evidence from field experiments in rural Mexico and Tanzania. Ecological Economics. 2012 Jan 15;73:220-7.

Krawczyk M. What brings your subjects to the lab? A field experiment. Experimental Economics. 2011 Nov;14:482-9.

Voslinsky A, Azar OH. Incentives in experimental economics. Journal of Behavioral and Experimental Economics. 2021 Aug 1;93:101706.

2. **Another problem is that the encouraging message about buying native plants was essentially the same for the control group and the treatment group, the latter differing only in a repeat and some extension of the message by the plant ecologist in person on video. So, was there really a substantive difference in treatment between the two groups?**

The goal of the experimental design was to determine whether a video expert testimonial had an impact on the pro-environmental behavior of purchasing native plants. To isolate this effect, participants in the control and treatment groups received the same information with the exception of the testimonial that was provided only to the treatment group. This set up provided for a clean identification of the impact of the testimonial because we varied only one feature of the information set provided to the two groups. Subjects were assigned to treatment and control groups randomly, which allowed us to estimate the causal effect of the testimonial treatment we were interested in isolating (Rubin, 1974).

Reference

Rubin DB. Estimating causal effects of treatments in randomized and nonrandomized studies. Journal of educational Psychology. 1974 Oct;66(5):688.

3. **A third problem may have resided in the unexpected invitation to participate in an experiment, while subjects had arrived with the intention (whatever) to attend the community event, to go shopping or to get an ice cream, respectively. It would seem that under such circumstances subjects being offered $ 10 for their (brief?) participation were naturally inclined to read instructions quickly and go along the presented questions soon enough to obtain their $ 10 reward, either or not lowered by the price of the bundle of plants they had purchased (or rather: had to purchase) following the study design, whereafter they would continue their journey in accordance with their original plan of visit.**

Subject recruitment for field experiments often takes place at venues that provide researchers with access to a diverse subject pool and real market environments (Canavari et al., 2019), such as the ones used to conduct our experiment. Participation in the experiment was voluntary and participants were informed of the time commitment associated with their participation. The experiment was administered by experienced researchers and trained administrators who sought to ensure that participants did not speed through the experiment and took the expected time in carrying out the tasks of the experiment.

References

Canavari M, Drichoutis AC, Lusk JL, Nayga Jr RM. How to run an experimental auction: A review of recent advances. European Review of Agricultural Economics. 2019 Dec 1;46(5):862-922.

**Some specific points**

a. **The title of the paper is rather general. A more specific and informative version would be: ”Encouraging consumers to buy native plants: Do expert testimonials about ecological benefits work?”**

Thank you for this suggestion. We prefer to keep a broader title for this paper because adoption of native plants in residential settings is a type of pro-environmental behavior. We have taken your comment into further consideration as we revised the abstract and introduction of the paper. In the revised manuscript, we are now more specific about the type of pro-environmental behaviors that are the focus of the research. Further, we believe that keeping the title as it currently stands will facilitate the ability of readers broadly looking for research related to pro-environmental behavior to find this paper via a general search as the implications are for more than just native plants.

b. **The first sentence of the Abstract (and the first paragraph of the Introduction..) needs qualification. In many situations much depends on the various costs (whatever) for a person or household to adopt pro-environmental behavior, and upon other encouragement strategies such as product availability, relevant knowledge and abilities, and the like. [The authors cite Abrahamse’s (2019) book, rich in empirical information about ‘what works, what doesn’t and why’.] The *last* sentence of the abstract provides non-surprising information which is unrelated to the difference between experimental conditions.**

Thank you for the suggestion. We have modified the first sentence of the abstract to clarify our focus on non-pecuniary interventions (expert testimonial, specifically) that may be effective at motivating pro-environmental behavior. While there are different factors that may drive the adoption of pro-environmental behavior, we felt that providing information about them in the introduction may be confusing to the readers as testing those factors is not the goal of this research. We edited the language of the abstract and the introduction to further clarify that this paper is focusing on non-monetary behavioral interventions.

Although the results stated in the last sentence of the abstract may be intuitive, we prefer to include them as they are part of the findings of this research, in addition to the primary results that the testimonial did not have a significant impact on participants’ WTP for native plants.

Abstract: “Using non-pecuniary interventions to motivate pro-environmental behavior appeals to program administrators seeking cost-effective ways to increase adoption of environmental practices. However, we should not expect that all interventions are effective and reporting when interventions fail is as important as documenting their successes. We used a framed field experiment with 308 adults from the Mid-Atlantic in the United States to test the effectiveness of an expert testimonial in encouraging adoption of native plants in residential settings. Though studies have found testimonials to be effective in other contexts, we find that the video testimonial had no effect on residents’ willingness to pay for native plants. Our analysis also shows that consumers who are younger, have higher incomes, and use other environmentally friendly practices on their lawns are more likely than other consumers to purchase native plants.”

[Page 2]: “Policymakers and program managers are increasingly interested in identifying effective interventions to promote pro-environmental behavior [1, 2]. Growing evidence suggests that non-pecuniary behavioral interventions have the potential to motivate energy and water conservation [3–8], lead to more environmentally friendly food choices [9], improve recycling efforts [10], and reduce nonpoint source pollution [11]. However, gaps remain in our understanding of the effectiveness of specific interventions in motivating pro-environmental behavior [12, 13]. Based on a review of 160 experiment-based studies of environmental interventions, Byerly et al. [12] identified areas in need of future research to inform the design of policies and programs, including testing the effectiveness of interventions used in other contexts in promoting pro-environmental behavior. Behavioral interventions such as information provision [14, 15], peer comparisons [16], social norms [17,18], and framing [19,20] have been extensively studied. Less is known about testimonials, such as opinions and recommendations, provided by experts and influencers who speak positively about a pro-environmental product or practice.”

c. **On p. 5, line 109, a phrase like “.. an incentive-compatible single-bounded dichotomous-choice framed field experiment” sounds unnecessarily complicated. Readers might want to know what ‘incentive-compatible’ and ‘single-bounded’ practically mean. And ‘binary’ seems simpler than ‘dichotomous’.**

We appreciate your concern. Much of this terminology is in-line with language common in experimental economics and is necessary to precisely describe the features of the experimental design. To help the reader, in the revised manuscript, we describe what incentive compatibility means as follows:

[Page 7]: “To ensure incentive-compatibility, participants were told that only one of the three purchase decisions would be binding, and that the binding decision would be randomly selected at the end of the study to determine participants’ take-home cash and whether they purchased the native plants. This mechanism ensured that participants’ dominant strategy was to answer ‘Yes’ whenever the posted price did not exceed their maximum WTP for the bundle.”

d. **Note that data collection for the reported experiment was in 2018, *i.e.* at least four years ago. Why such delay?**

We appreciate the concern and we would have preferred to have published this paper immediately after the data was collected. However, there were elements that delayed the final write-up of the paper, including the COVID pandemic. Ultimately, the time lag between data collection and publication had no impact on the quality of the research or this publication.

e. **On p. 6, lines 122-123, sentence “Participants completed the experiment asynchronously and were not permitted to communicate with other participants present at the same time” raises questions about the practical setting in which subjects had to perform the requested task. See also general problem 3 above.**

The experiment was administered by a team of trained administrators who ensured that all participants were following the same protocols. At each venue where the experiment took place, the research team set up tables with chairs for the participants and provided a display of the plants participants would be able to purchase. Each participant who voluntarily agreed to participate in the experiment was seated at an individual table and was provided an iPad to complete the experiment. The team of researchers and administrators who were running the experiment ensured that participants completed the experiment on their own, did not rush through the experiment, and answered any questions that participants had. Ensuring that each participant completed the experiment on their own was an important part of the experimental design so that we could collect individual preferences unaffected by the opinions of others. While allowing communication between participants would have been an option, we felt that our research design was cleaner by not permitting communication as we did not want spillover effects, such as when a person in the testimonial treatment talks about that information to a person in the control treatment.

f. **Also on p. 6, lines 141-142: the three-point WTP-scale of $ 0, 3 and 6 may not have been sensitive or extensive enough to assess the possible added effects of the video-message following the written instruction.**

This is an interesting comment. Future research should explore the sensitivity of WTP to price scale.

g. **A nice aspect of the paper is the authors’ exercise about the (in-) sufficiency of statistical power to detect possible treatment effects. On p. 15, lines 284-285, the authors conclude that “Based on our power analyses, we cannot rule out the possibility that the testimonial has [or rather: ‘had’?] an effect below the $0.96 to $1.50 range.” If so, this might have meant that subjects were willing to pay (approximately) fifty cents more in response to the video-message. Would the authors have found this an interesting result worth publishing in a professional journal? [And note again general problem 2 above.]**

We are being transparent about the size of effect this experiment was powered to detect. Given the statistical power of our experiment, we do not find that an expert testimonial has a significant impact on participants’ WTP. This finding is worth publishing as more robust literature is needed that not only identifies what types of behavioral interventions work, but importantly also identifies things that do not work or have limited impacts. Future research can explore smaller effect sizes with great sample sizes. However, researchers must weigh the costs of larger sample sizes against the potential benefits of detecting smaller effects. If our findings indicated that subjects were willing to pay fifty cents more in response to the video-message, we would have found this result worth publishing.

h. **Experienced researchers in ‘encouraging pro-environmental behavior‘ might object to the strong generalization speaking from the passage on p. 16, lines 307-310: “Contrary to studies of testimonials used in other contexts […], we find that the expert’s video testimonial had no significant identifiable effect on WTP for native plants. Consequently, in pro-environmental contexts, testimonial-based interventions alone are not necessarily sufficient to motivate changes in behavior.” The generality of this conclusion does not seem entirely warranted.**

The goal of this statement is to summarize our findings that are in contrast to the results found in other contexts. We employ a strong experimental design and are transparent about the size of the effects that can be detected in our experiment. We edited the last sentence of this passage to make this statement softer:

[Page 17]: “Consequently, in pro-environmental contexts, testimonial-based interventions alone may not necessarily be sufficient to motivate changes in behavior.”

It is not clear what is implied by the statement “experienced researchers in encouraging pro-environmental behavior.” The team of researchers that conducted this study has significant experience in researching a wide range of topics related to environmental behavior and environmental policies and has substantial experience in designing economic experiments.
